# Clinical and pathological features of bronchiolitis obliterans requiring lung transplantation in paraneoplastic pemphigus associated with Castleman disease

**DOI:** 10.1111/crj.13465

**Published:** 2022-01-21

**Authors:** Wenhui Chen, Ling Zhao, Lijuan Guo, Li Zhao, Hongtao Niu, Huifang Lian, Huaping Dai, Jingyu Chen, Chen Wang

**Affiliations:** ^1^ Department of Lung Transplantation Centre of Respiratory Diseases, China‐Japan Friendship Hospital Beijing China; ^2^ National Center for Respiratory Medicine Beijing China; ^3^ Institute of Respiratory Medicine, Chinese Academy of Medical Science Beijing China; ^4^ National Clinical Research Center for Respiratory Diseases Beijing China; ^5^ Department of Pathology Centre of Respiratory Diseases, China‐Japan Friendship Hospital Beijing China; ^6^ Department of Pulmonary and Critical Care Medicine Centre of Respiratory Diseases, China‐Japan Friendship Hospital Beijing China; ^7^ Department of Pulmonary and Critical Care Medicine Jizhong Energy Fengfeng Group Hospital Handan China; ^8^ WHO Collaborating Center for Tobacco Cessation and Respiratory Diseases Prevention Beijing China

**Keywords:** bronchiolitis obliterans, Castleman disease, lung transplant, paraneoplastic pemphigus

## Abstract

**Summary at a glance:**

Bronchiolitis obliterans in paraneoplastic pemphigus associated with Castleman disease possesses the progressive nature even when it is treated with intensive medical therapy. Antibodies were at least in low titers before the Lung transplant and remain negative after the procedure. Explanted lungs showed coexistence of cellular destructive bronchiolitis and constrictive bronchiolitis.

**Background:**

Bronchiolitis obliterans (BO) in paraneoplastic pemphigus (PNP) associated with Castleman disease (CD) possesses the progressive nature of pulmonary disease even when it is treated with intensive medical therapy. Lung transplantation (LT) offers an acceptable form of treatment.

**Methods:**

We conducted a retrospective study of two cases of BO in PNP associated with CD who underwent LT between March 2017 and March 2020 at the China‐Japan Friendship Hospital. We also included one case from the literature.

**Results:**

In this patient series, PNP was the primary clinical presentation in all patients, and it was accompanied by respiratory symptoms before/after CD excision. In spite of being treated with various combinations of immunosuppressive and anti‐inflammatory agents, the patients had great or total improvement in mucosal erosions, whereas their pulmonary function test (PFT) deteriorated gradually or sharply. The duration times from disease onset to timing of LT were 1, 2 and 5 years. All antibodies were negative or were present at low titers before the LT procedure and remain negative after the procedure. The histopathological features of explanted lungs showed cellular and coexistent destructive bronchiolitis and constrictive bronchiolitis in two cases. Granulation with numerous foamy macrophages, scattered giant cells and cholesterol clefts were especially prominent in case one.

**Conclusion:**

BO in PNP associated with CD had poor clinical outcomes. LT was preferable choice in end‐stage BO when PNP and CD were controlled.

## 1 INTRODUCTION

Castleman disease (CD) is a distinct lymphoproliferative disorder of uncertain origin and is highly heterogeneous. Paraneoplastic pemphigus (PNP) is an example of a paraneoplastic phenomenon caused by an autoimmune disease initiated by an underlying lymphoproliferative disorder.^1^ A systematic review demonstrated that CD as a primary tumour in patients with both mucocutaneous and respiratory complications of PNP occurred in 71.2% of patients, which was followed by lymphoma (18.6%).^2^ Bronchiolitis obliterans (BO) is frequently found in PNP, with incidence rates ranging from 27% to 93%, and may cause respiratory failure and death.^3^, ^4^, ^5^, ^6^, ^7^, ^8^ The patients consistently do not respond to intensive medical therapy, and no therapy can control the progression of this pulmonary disease.^9^, ^10^, ^11^

Given the progressive nature of the pulmonary disease in spite of intensive medical therapy, lung transplantation (LT) offers an acceptable form of treatment. To better understand the respiratory complications accompanying PNP associated with CD and to explore a rational regimen to reduce the high mortality of this disorder, we analysed the characteristics of two LT cases in our hospital and one case from literature.

## 2 MATERIALS AND METHODS

### 2.1 Study population

#### 2.1.1 Our cases

This retrospective study was conducted at China‐Japan Friendship Hospital (CJFH) and was approved by the CJFH Ethics Committee (number 2019‐164‐K113).

A total of 258 lung transplantation patients received lung transplants in our centre from March 2017 to March 2020. We included all cases with BO caused by lung involvement in CD patients.

#### 2.1.2 Cases from the literature

In addition to our cases, we searched the English‐language published literature using PubMed/MEDLINE with the search terms ‘Castleman disease’ and/or ‘paraneoplastic pemphigus’ and/or ‘bronchiolitis obliterans’ and ‘lung transplantation’ from 1990 to 2020.

#### 2.1.3 Definitions

Clinical diagnosis of CD and complications were established following generally accepted guidelines.^12^, ^13^ Multicentric CD (MCD) was defined by the involvement of ≥2 lymph nodes or regions. The remaining cases were classified as Unicentric CD (UCD).

PNP was diagnosed based on the minimal criteria proposed by Anhalt.^1^ These criteria included progressive stomatitis; acantholysis, lichenoid, or interface dermatitis in cutaneous or oral histology; serum anti‐plakin autoantibodies detected by immunoblotting or immunoprecipitation; and underlying lymphoproliferative neoplasm.

### 2.2 Data collection and analysis

A standard form was used to record the characteristics of the patients. The form consisted of the following items: (1) demographic data; (2) characteristics of mucocutaneous and respiratory manifestations, including pulmonary functional and high‐resolution computed tomography (HRCT); (3) laboratory and immunologic features; (4) LT procedures and outcomes; and (5) histopathological features of explanted lungs.

For indirect immunofluorescence, frozen sections of rat urinary bladder were used as the substrates, and the patients' serum was used as the primary antibodies. An enzyme‐linked immunosorbent assay (ELISA) test kit was used to assay anti‐desmogleins 1 and 3 (Dsg1 and Dsg3) IgG in patients' serum with cut‐off index values of 20 for Dsg1 and Dsg3.^4^

## 3 RESULTS

### 3.1 Case one

A 22‐year‐old man presented with ulcerating bullous lesions in the oral and genital regions and conjunctivitis with weight loss of 10 kg 2 years before LT. PNP was diagnosed, and positron emission tomography‐CT (PET‐CT) revealed 1.6 × 1.4 × 0.9‐cm soft tissue pelvic mass with a high standard uptake value (SUV). The pelvic nodular mass was completely resected to reveal HHV‐8‐negative unicentric/hyaline vascular (HV) variant CD with intravenous administration of immunoglobulin before the operation.

Whereas mucosal erosions improved after resection of UCD, one month after the operation, he was admitted to the hospital due to a complaint of dyspnoea on exertion. In spite of treatments with prednisone, thalidomide and intravenous immunoglobulin and following budesonide/formoterol azathioprine, montelukast and tiotropium administration, he experienced increasing progressive dyspnoea on exertion. He presented to the hospital frequently with recurrent onset of respiratory infection with carbapenem‐resistant *Acinetobacter baumannii* (CRAB) and *Aspergillus fumigatus*.

He experienced hypercapnic respiratory failure with tachycardia and required the assistance of a noninvasive ventilator when he was admitted to our hospital for referral for LT.

After a difficult 2‐month wait on the waiting list, he underwent sequential bilateral LT (BLT) in May 2019. He experienced an uneven postoperation time with primary graft dysfunction (PGD), severe bronchitis with CRAB infection and anastomotic stenosis, but he has now returned to his normal life.

### 3.2 Case two

A 60‐year‐old man presented with painful stomatitis, ulcerating bullous lesions on the tongue and dystrophic nails, which were suggestive of lichen planus, 5 years before LT. Subsequently, he developed dry cough and dyspnoea on exertion. Abdominal enhanced CT showed a retroperitoneal 9 × 6 × 7‐cm soft tissue mass. The retroperitoneal mass was completely resected to reveal unicentric/mixed cellular variant CD. Postoperatively, over a 5‐year period, the patient was treated with various combinations of immunosuppressive and anti‐inflammatory agents, including high‐dose steroids, thalidomide, and methotrexate. He experienced dramatic improvement of his mucosal erosions, which correlated with a marked decrease in circulating antidesmoplakin antibody titers. Unfortunately, his respiratory symptom progressed to the point that it severely limited his activities, and he required supplemental oxygenation and the assistance of a noninvasive ventilator intermittently in spite of combined treatment with salmeterol/fluticasone, azathioprine, montelukast, and tiotropium. During this period, he had recurrent episodes of respiratory infection with carbapenem‐resistant *Pseudomonas aeruginosa* (CRPA) and had a weight loss of 25 kg.

After a comprehensive evaluation, he underwent BLT in October 2019. He experienced bronchial ischaemia and necrosis accompanied by severe CRPA infection and the following bronchial stenosis. He is now slowly recovering.

**FIGURE 1:**
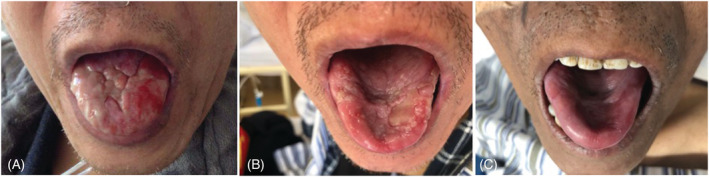
(A–C) In case two, the ulcerative lesions on the tongue 9 months after Castleman disease (CD) excision (A), before lung transplantation (LT) operation (B), and 2 months after LT operation (C)

His ulcerative lesions on the tongue changed during the examination period (Figure 1).

**FIGURE 2:**
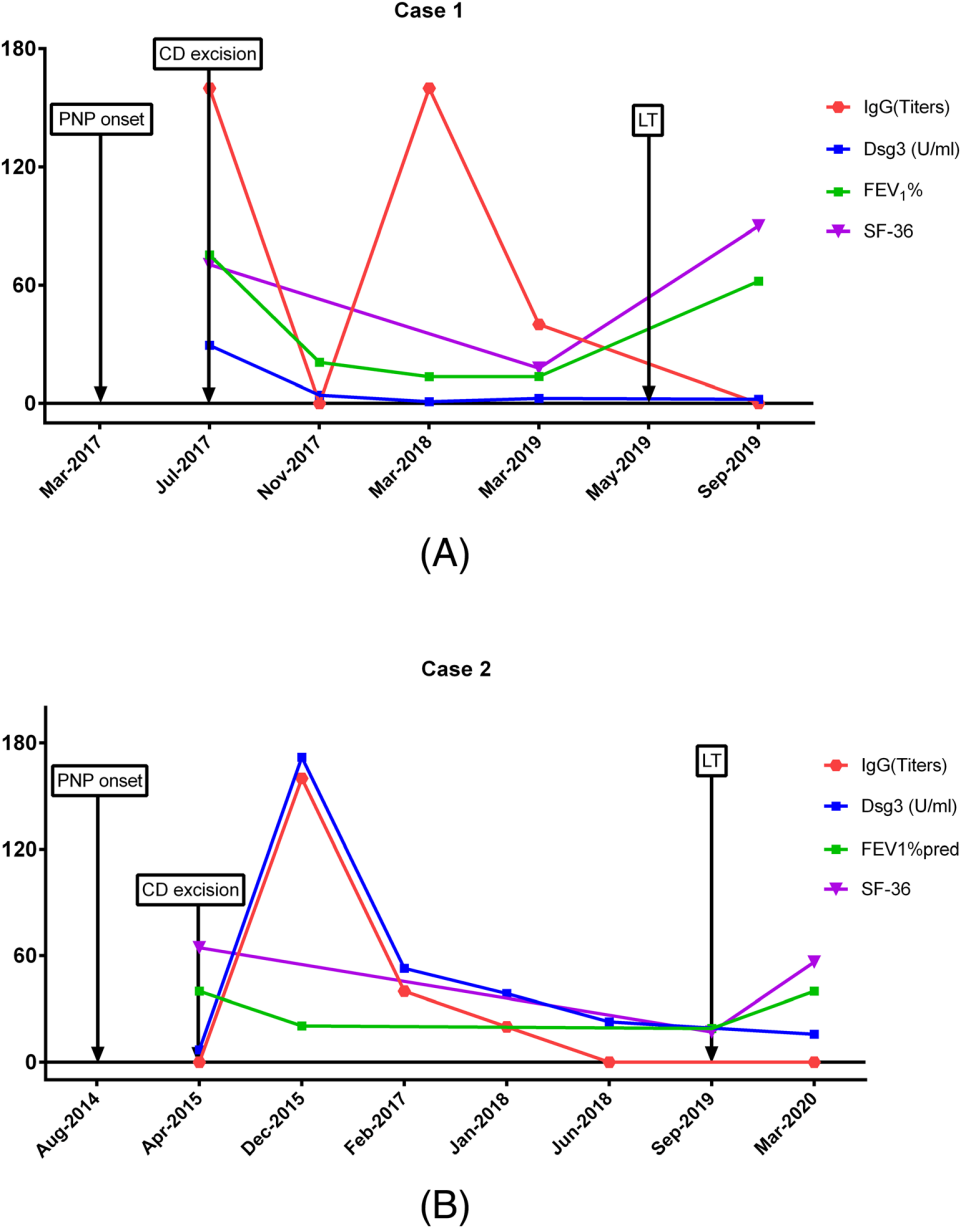
The medical history, dynamic curve of FEV1, short form 36 (SF‐36) results and autoantibody information for the two patients (2A: case one, 2B: case two)

Health‐related quality of life (HRQL), measured by the physical health component summary of the short form 36 (SF‐36) questionnaire, was evaluated in our patients. They all had great improvement of HRQL, especially in case one (Figure 2A,B).

### 3.3 Case from the literature

We identified only one case with BO complicated with PNP who underwent LT in CD.^14^ The details of the clinical features and outcomes are presented in Table 1.

**TABLE 1:** Characteristics of clinical manifestation of CD in present study and from literature

Patient No	Sex	Age	Mucosal leisions			conjunctivitis	skin	Location of CD	Type of CD onset to LT (years)	CD size (cm)	TIME (from disease onset to LT)
			Oral	Ocular	genital						
1	M	22	+	+	+	+	−	Pel	HV/UCD	1.6 × 1.4 × 0.9	2y
2	M	60	+	+	+	−	+	Ret	Mixed/UCD	9 × 6 × 7	5y
3	M	14	+	+	+	+	−	Med	HV/UCD	8 × 6 × 2.5	1y

Abbreviations: M: male; CD: Castleman disease; HV: hyaline vascular; Pel: pelvic; UCD: unicentric Castleman disease; Ret: retroperitoneum; Med: mediastinum; LT: lung transplant；+: present; −: absent.

### 3.4 Immunologic features

Indirect rat bladder antibody testing by indirect immunofluorescence was positive at a dilution of 1:160. IgG ELISA for Dsg3 was positive at 29.39 U/ml at the beginning of disease onset in case one. Indirect rat bladder antibody testing by indirect immunofluorescence was positive at a dilution of 1:10. IgG ELISA for Dsg3 was positive at 31.31 U/ml in case two.

Interleukin‐6 (IL‐6) levels increased to 10.4 ng/ml (0–5.9 ng/ml) before the LT procedure and returned to normal after the operation in case two, while IL‐6 levels were normal before and after the operation in case one.

### 3.5 Pulmonary function tests and Hhigh‐resolution computed tomography findings

**FIGURE 3:**
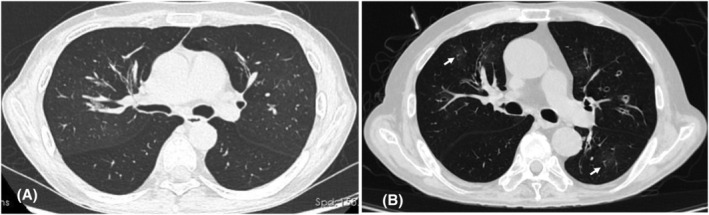
(A) Case 1 high‐resolution computed tomography (HRCT) showed mild, diffuse bronchial wall thickening. (B) Case 2 HRCT presented mild, diffuse bronchial wall thickening with mild bronchiolectasis and patchy centrilobular ground‐glass opacities (arrow)

In case one, the patient referred to LT evaluation in Jan 2019 (3 months before LT). PFT showed forced expiratory volume in one second (FEV1) of 13.5%, forced vital capacity (FVC) of 29.6% (FEV1/FVC ratio, 38.37%) which worsened sharply after one and a half years of CD excision. HRCT showed mild, diffuse bronchial wall thickening (Figure 3A). One month before LT procedure (Sep 2019) in case two, PFT showed FEV1 of 20%, FVC of 43% (FEV1/FVC ratio, 36.65%) which worsened gradually in the following 5 years after CD excision. HRCT presented mild, diffuse bronchial wall thickening with mild bronchiolectasis and patchy centrilobular ground‐glass opacities (Figure 3B).

### 3.6 Histopathological features of explanted lungs

**FIGURE 4:**
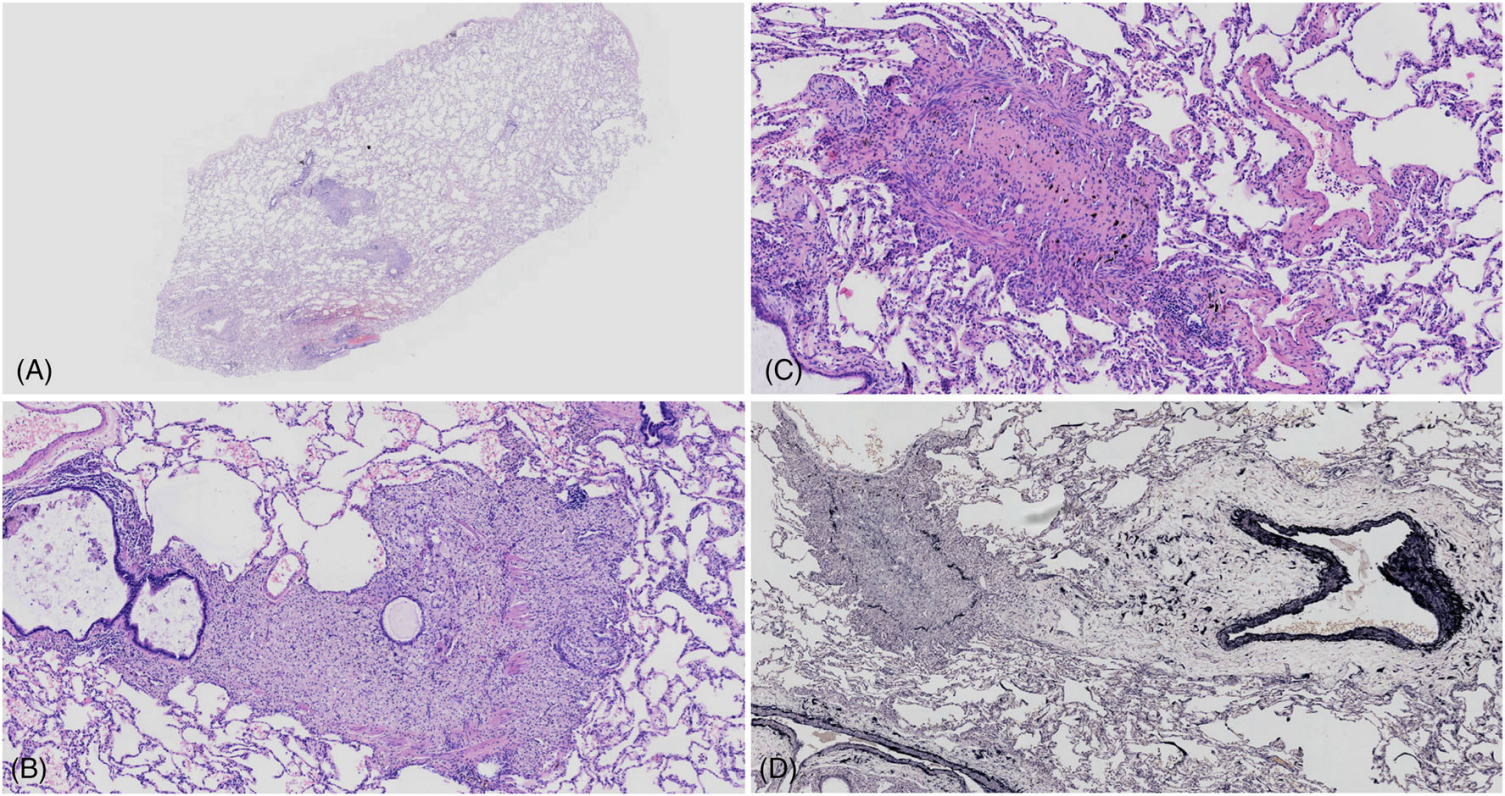
Case 1: (A) Low‐magnification microscopic appearance of the left lower bronchiole. Lesions are exclusively limited to the area of the membranous bronchiole (original magnification ×40). (B) Granulation with plenty of foamy macrophages, with scattered giant cells causing stenosis of the bronchiolar lumen, whereas smooth muscle of the wall was partially destroyed. Epithelial sloughing and mucous retention are seen in the lumen (original magnification ×200). (C) Complete obliteration of the bronchial lumen due to submucosal concentric fibrosis (original magnification ×200). (D) There is total fibrous obliteration of the lumen (original magnification ×100). (A–C) Haematoxylin‐Eosin stain; (D) Elastic van Gieson stain

Case one: Granulation with plenty of foamy macrophages, scattered giant cells and cholesterol clefts caused stenosis of the bronchiolar lumen, whereas the smooth muscle of the wall was partially destroyed. There was subtotal or total fibrous obliteration of the lumen with plenty of lymphoplasmacytic cells infiltrating the airway wall. These findings were compatible with constrictive bronchiolitis. Necrosis and partial denudation of the epithelium were present. Emphysema and bullae with rupture of alveolar septum can be seen. We detected focal fibrinous exudates, cholesterol clefts and macrophages at the alveoli (Figure 4A–D).

**FIGURE 5:**
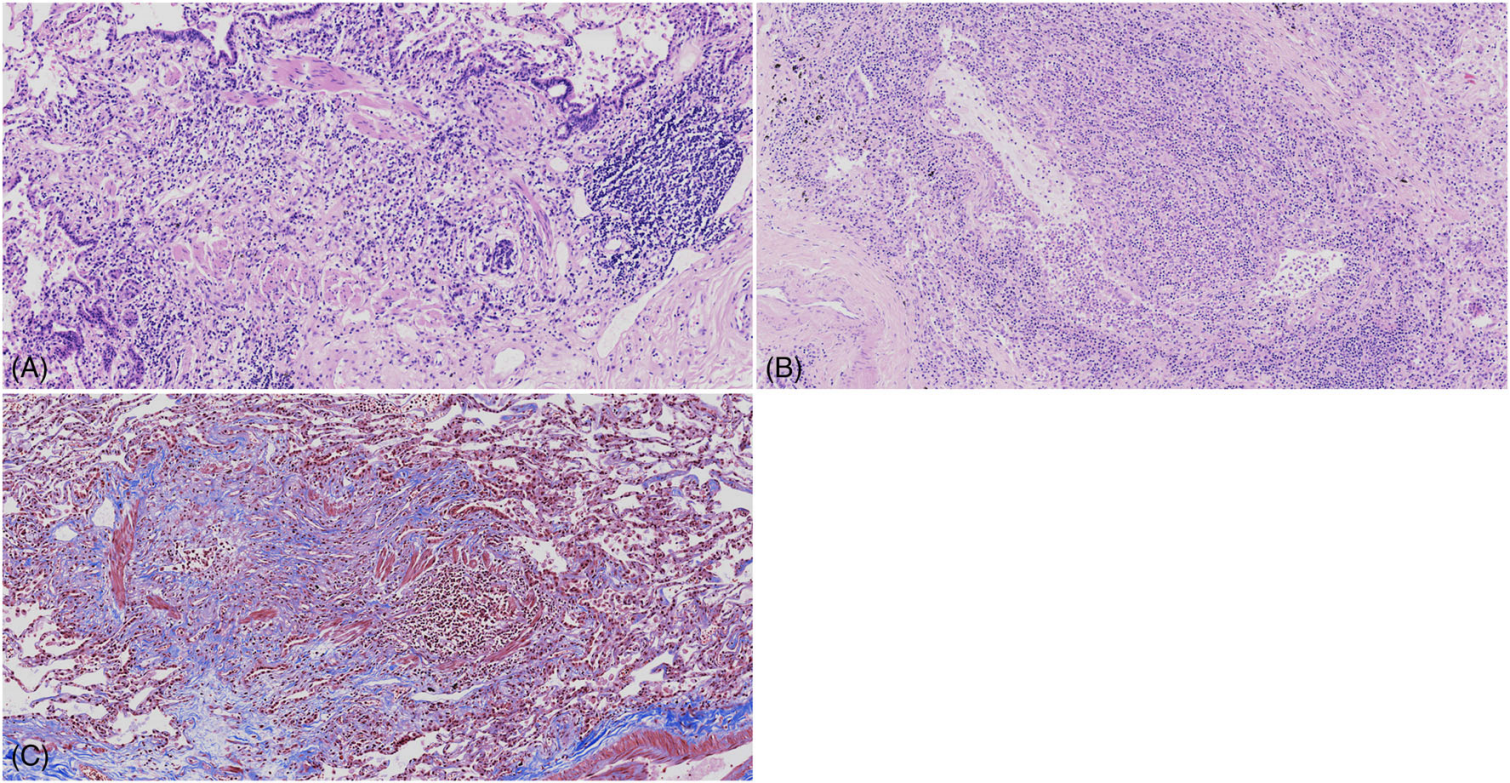
Case 2: (A) Granulation with numerous inflammatory cells caused complete obliteration of the bronchiolar lumen, whereas the smooth muscle of the wall was partially destroyed (original magnification ×200). (B) There was many lymphoplasmacytic and neutrophilic cells that infiltrated the bronchial lumen and walls associated with patchy areas of acantholytic epithelial detachment and abundant mucus in the airways (original magnification ×200). (C) Granulation with plenty of inflammatory cells obliterate the bronchial lumen whereas smooth muscle of the wall was partially destroyed. (A,B) Haematoxylin‐Eosin stain. (C) Masson's trichrome stain

Case two: Compared to case one, there were much less granulation with foamy macrophages and more prominent lymphoplasmacytic and neutrophilic cells infiltrating the bronchial lumen and wall. There was extensive squamous metaplasia of the proximal airways associated with patchy areas of acantholytic epithelial detachment, basement‐membrane thickening, and abundant mucus in the airways (Figure 5A–D).

## 4 DISCUSSION

### 4.1 Clinical features

Clinically, CD is characterized as UCD and MCD. Pathologically, CD can be classified into three variants: HV, plasmacytic variant (PC) and mixed cellular variant. Distinct clinical features and prognoses have been reported in some patients with PNP associated with CD, which is usually classified as UCD or HV.^5^, ^15^, ^16^, ^17^ Our consecutive two cases and one case from the literature were all classified as UCD, with two of them being HV, while the other one was Mix.

In this patient series, PNP was the primary clinical presentation in all patients, accompanied by respiratory symptoms before/after CD excision. In spite of being treated with various combinations of immunosuppressive and anti‐inflammatory agents, they all had great or total improvement in mucosal erosions, whereas their PFT deteriorated gradually or sharply. The duration times from disease onset to timing of LT were 1, 2 and 5 years, respectively. The presence of PNP is known to be the most significant adverse prognostic factor among CD patients, with a 3‐year mortality of greater than 40% as opposed to 12% among those without PNP.^5^ In Anhalt's experience with 84 patients with PNP, the mortality rate was more than 90%. A recognized complication in approximately 30% of patients is respiratory failure with features of BO.^18^

### 4.2 Immunological and histopathologic features

In our two cases, indirect immunofluorescence assay on rat bladder tissue of PNP patients revealed maximum titers of 1:160 and positivity at a dilution of 1:320, and anti‐Dsg 3 antibody levels were 29.39 and 280.21 U/ml. Their autoantibody values changed after CD excision, though the change did not parallel with PNP. All antibodies became negative after the LT procedure. Our case two and the case from the literature had elevated IL‐6 levels before LT, while all patients had normal levels after LT.

IL‐6 plays a fundamental role in the pathogenesis of CD. IL‐6 promotes B cell differentiation and drives immunoglobulin production, and dysregulated IL‐6 production has been implicated in specific autoimmune diseases. Castleman's patients have also been shown to have high serum levels of IL‐6.^1^ However, anti‐IL‐6 therapies such as tocilizumab fail to prevent the development of BO with CD‐associated PNP.^11^ Next, we can use explanted lungs to test autoantibodies and IL‐6 levels and try to further define the mechanism of PNP‐associated BO.

Histopathologically, BO is mainly classified into two categories: cellular and destructive bronchiolitis (CDB). The narrowing of the airway lumen is due to intraluminal, mural, and peribronchiolar infiltration of inflammatory cells as well as proliferation of granulation tissue, which destroys the normal structural components, including elastic fibre and smooth muscle; constrictive bronchiolitis (CoB) is characterized by concentric narrowing of the airway lumen due to submucosal fibrosis.^19^, ^20^

The bronchiolar lesions are patchy, even in severely affected patients, and they may be missed unless serial section and trichrome stains (to appreciate the fibrotic component) are performed.^21^, ^22^ Sugino et al. demonstrated that most CoB appeared to be a distinct process independent of CDB and considered that these two morphologic subtypes were not coexistent.^20^

However, our cases tell a different story. CDB and CoB were coexistent in the two cases. Granulation with plenty of foamy macrophages, scattered giant cells and cholesterol clefts were especially more prominent in case one. We speculate that acute and/or chronic respiratory infections in addition to an abnormality in the systemic immune response may cause the two different phenotypes of BO such as CDB and CoB. PNP is a complicated autoimmune disease, while the mechanism of PNP‐associated BO may be due to not only autoantibodies but also CD8+ T lymphocytes, which may have a key role in the progression of the bronchiolitis.^23^ Autoantibody‐mediated injury takes an important role in the pathogenesis of BO, although infections and toxic effects induced by chemotherapy and neoplasm may also cause pulmonary injury.^3^, ^23^, ^24^ Autoantibodies directed against plakin proteins may be responsible for acantholytic changes in the respiratory epithelium.^18^

### 4.3 Timing and outcome of lung transplantation for bronchiolitis obliterans in Castleman disease‐associated paraneoplastic pemphigus

Regarding the timing of an LT in BO patients, there were no specific guidelines for lung transplantation candidate selection. In BO or other Late‐onset noninfectious pulmonary complications post‐SCT, 17 out of 20 centres (85%) reported adapting the ISHLT consensus for cystic fibrosis.^25^, ^26^

However, owing to its own unique characteristics, the need to gain control of the paraneoplastic autoimmune manifestations of this disease (as measured by a decrease in circulating autoantibodies) is of critical importance before LT. Our two patients had low body‐mass index (BMI 14.8–16.4 kg/m^2^), frequent multidrug resistant bacterial infections (CRAB, CRPA) during treatments with various of immunosuppressive agents and low ability for pulmonary rehabilitation, which all increased the difficulty for LT perioperative management.

They all had great improvement of HRQL which was measured by SF‐36 questionnaire, especially in case one. In recent years, increasing attention has been given to assessments of the quality of survival, with particular importance given to the patients' self‐evaluations of HRQL after the transplant procedure.^27^, ^28^

This study had several limitations that should be discussed. Firstly, due to the small case series, we are unable draw a comprehensive picture of characteristics of PNP‐associated BO. Secondly, to compare the difference between PNP‐associated BO with other underlying diseases such as connective tissue disease, graft versus host disease may help in further understanding the nature of the disease.

## CONFLICT OF INTEREST

The authors have declared no conflicts of interest.

## AUTHOR CONTRIBUTIONS

Chen Wang, Jingyu Chen, Huaping Dai designed the research; Whenhui Chen wrote the paper; Lijuan Guo, Li Zhao, Chaoyang Liang, Lei Jing contribute to patients' follow‐up and data collection; Hongtao Liu analyse the data; Ling Zhao reviewed the pathological data.

## ETHICS STATEMENT

The ethical approval form has been provided.

## Data Availability

The data that support the findings of this study are available on request from the corresponding author. The data are not publicly available due to privacy or ethical restrictions.
